# Ferroptosis and EMT resistance in cancer: a comprehensive review of the interplay

**DOI:** 10.3389/fonc.2024.1344290

**Published:** 2024-02-26

**Authors:** Huiming Zhang, Naifeng Chen, Chenglong Ding, Huinan Zhang, Dejiang Liu, Shuang Liu

**Affiliations:** ^1^ School of Basic Medicine, Jiamusi University, Jiamusi, China; ^2^ College of Biology and Agriculture, Jiamusi University, Jiamusi, China

**Keywords:** ferroptosis, metastasis, tumorigenesis, drug resistance, cancer therapeutics

## Abstract

Ferroptosis differs from traditional cell death mechanisms like apoptosis, necrosis, and autophagy, primarily due to its reliance on iron metabolism and the loss of glutathione peroxidase activity, leading to lipid peroxidation and cell death. The dysregulation of iron metabolism is a hallmark of various cancers, contributing to tumor progression, metastasis, and notably, drug resistance. The acquisition of mesenchymal characteristics by epithelial cells is known as Epithelial–Mesenchymal Transition (EMT), a biological process intricately linked to cancer development, promoting traits such as invasiveness, metastasis, and resistance to therapeutic interventions. EMT plays a pivotal role in cancer progression and contributes significantly to the complex dynamics of carcinogenesis. Research findings indicate that mesenchymal cancer cells exhibit greater susceptibility to ferroptosis compared to their epithelial counterparts. The induction of ferroptosis becomes more effective in eliminating drug-resistant cancer cells during the process of EMT. The interplay between ferroptosis and EMT, a process where epithelial cells transform into mobile mesenchymal cells, is crucial in understanding cancer progression. EMT is associated with increased cancer metastasis and drug resistance. The review delves into how ferroptosis and EMT influence each other, highlighting the role of key proteins like GPX4, which protects against lipid peroxidation, and its inhibition can induce ferroptosis. Conversely, increased GPX4 expression is linked to heightened resistance to ferroptosis in cancer cells. Moreover, the review discusses the implications of EMT-induced transcription factors such as Snail, Zeb1, and Twist in modulating the sensitivity of tumor cells to ferroptosis, thereby affecting drug resistance and cancer treatment outcomes. Targeting the ferroptosis pathway offers a promising therapeutic strategy, particularly for tumors resistant to conventional treatments. The induction of ferroptosis in these cells could potentially overcome drug resistance. However, translating these findings into clinical practice presents challenges, including understanding the precise mechanisms of ferroptosis induction, identifying predictive biomarkers, and optimizing combination therapies. The review underscores the need for further research to unravel the complex interactions between ferroptosis, EMT, and drug resistance in cancer. This could lead to the development of more effective, targeted cancer treatments, particularly for drug-resistant tumors, offering new hope in cancer therapeutics.

## 1 Introduction

Cells are the basic units of living organisms, and normal cell proliferation, differentiation, and apoptosis are cycles of life activities. However, tumor cells differ from normal cells in that they can bypass the entire apoptosis process, proliferate and differentiate indefinitely, thereby impacting life and health (1). The various cell death processes can be divided into normal apoptosis, cell thermosis, or cell necrosis. Different death categories have their lethality mechanisms (2). Ferroptosis is a non-apoptotic form of cell death first defined in 2012. Erastin, unlike many inducers, acts on various molecular structures to increase ROS levels in the mitochondrial respiratory chain of cells. In most organisms, oxygen typically serves as the primary electron acceptor in redox metabolism. Cells generate energy through electron transfer in redox reactions and maintain normal cellular functions. There are many causes of cellular oxidative stress, and peroxidation is one of the important regulators, via a process known as ferroptosis (3). This factor can lead to phospholipid peroxidation and ferroptosis (4). Ferroptosis is regulated by various cell metabolic pathways, including iron metabolism, mitochondrial activities, and amino acids, among others. In addition, redox imbalances are among the causes of many diseases (5). Iron deficiency is an important indicator of redox imbalance, and it is also closely associated with a series of tumor cell-associated pathological changes (6).

The p53 protein is an important tumor suppressor and is the most common mutated gene in human cancer. In addition to losing its cancer-suppressing functions, p53 mutation exerts carcinogenic effects. Inactivation of p53 and ferroptosis are also related. The p53 protein regulates cell division, inhibits abnormal division of mutated cells, and prevents tumor cell formation by sending apoptosis signals to such cells. During cancer formation, p53 coordinates multiple responses and enhances ferroptosis by inhibiting SLC7A11 expressions. Inhibition of activities of the cystine/glutamate transporter system decreases cystine absorption, thereby reducing GSH synthesis, followed by decreased membrane lipid repair enzyme GPX4 activities, reduced antioxidant capacities and ferroptosis (7). Glutathione is a ubiquitous low molecular weight antioxidant in cells whose homeostasis is mainly dependent on required amino acid precursor absorption rates, biological activities of related enzymes, and redox balance. The loss of p53, mutation of proto-oncogenes and overexpressions of the tumor-promoting functional protein (OTUB1) increases SLC7A11 levels, which inhibits ferroptosis to accelerate tumor growth (8). Cancer cells with high expressions of SLC7A11 are more sensitive to glutamine and glucose starvation, which accelerates metabolic processes. Targeting carbonic anhydrase (CAIX) protects cancer cells from ferroptosis-induced death. Combining the inhibitors of CAIX with compounds known to cause ferroptosis can lead to cancer cell death. Once CAIX activities are inhibited, it leads to acidification of the environment and accelerates the accumulation of reactive oxygen species in cells. Therefore, the alkaline environment in cells is of far-reaching significance in inhibiting ferroptosis and improving tumor drug resistance (9).

Chemotherapy is the preferred therapeutic option for cancer, however, long-term chemotherapy leads to increased tumor cell resistance (10). Ferroptosis is associated with cancer drug resistance. Mesenchymal stem cells (MSCs) play a key role in drug resistance (11). Highly mesenchymal stem cells confer drug-resistant properties to different cancer types. A growing body of clinical evidence suggests that targeting ferroptosis holds promise as a potential strategy to overcome drug resistance and augment the therapeutic effectiveness of anticancer treatments (12). Ferroptosis inducers demonstrate the ability to reverse acquired resistance in cancer cells to various anticancer agents such as lapatinib, cisplatin, docetaxel, sorafenib, among others. Inhibiting xCT and GPX4 has been identified as a mechanism to induce cancer cell death in response to conventional chemotherapy or radiotherapy (13). Specifically, xCT inhibition enhances cancer cell sensitivity to anticancer agents by depleting intracellular glutathione (GSH) through the blockage of cystine uptake (14). The adoption of a high-mesenchymal cell state has been associated with reduced sensitivity across various cancer cell types. Notably, the therapy-resistant high-mesenchymal cell state has been implicated in evading ferroptosis by regulating lipid peroxidation. Inhibition of GPX4 triggers peroxide reactions mediated by intracellular iron, culminating in ferroptosis (15). Consequently, inducing ferroptosis emerges as an effective strategy to eliminate the high-mesenchymal cell state in cancer cells.We systematically describe the mechanisms of action of ferroptosis and introduce the effects of ferroptosis in reversing cancer drug resistance. This paper also focuses on the feasibility and the challenges associated with new cancer drug treatment approaches.

## 2 Ferroptosis mechanisms

Studies are increasingly aimed at elucidating the mechanisms of ferroptosis. The impact of cellular metabolism as well as the associations between ferroptosis and cellular metabolic pathways have been investigated. Ferroptosis is caused by imbalanced production and degradation of lipid reactive oxygen species in cells. Elevated lipid reactive oxygen species levels have been shown to result in oxidative cell death, a process referred to as ferroptosis. This process is characterized by; lipid peroxidation, decreased glutathione peroxidase (GPXs) activities, increased ROS levels in tumor cells (16), and wrinkled cell membrane without significant changes of the nucleus (17).

**Figure 1:**
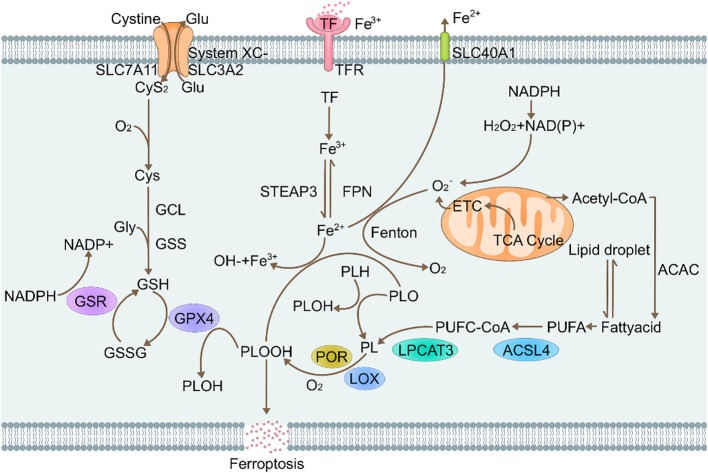
Molecular mechanisms of ferroptosis. Glu, Glutamate; SLC7A11, Recombinant Solute Carrier Family 7, Member 11; CyS2, cysteine2; O2, oxygen; Gcl, Glutamate Cysteine ligase; Gly, glycine; GSS, glutathione synthetase; GSR, glutathione reductase; GSH, L-glutathione; NADPH, nicotinamide adenine dinucleotide phosphate; GSSG, Glutathione(Oxidized); GPX4, glutathione peroxidase 4 (phospholipid hydroperoxidase); PLOOH, Phospholipid hydroperoxide; Lox, lipoxygenases; Lpcat3, Recombinant Lysophosphatidylcholine Acyltransferase 3; STEAP3, six-transmembrane epithelial antigen of prostate 3; TF, transferrin; FPN, recombinant ferroportin; SLC40A1, solute carrier family 40 (iron-regulated transporter), member 1; ACSL4, acyl-CoA synthetase long-chain family member 4.

Ferroptosis symptoms are associated with: i. GPX4 enzymes; ii. Phospholipid peroxidation and iii. Iron metabolism regulatory pathways (**Figure 1**).

### 2.1 Regulation of the GPX 4 regulatory pathway

Cells are affected by multiple signal-regulating channels, such as apoptosis, necrotic and ferrotic channels. Among the regulatory pathways, ferroptosis is the most specific form, Due to the accumulation of intracellular iron ions, lipid peroxidation reaction occurs (18). Since GPX4 is a regulator of the nephrotic core, it can be used to determine the occurrence of ferroptosis in cells. The mitochondria shrink due to ferroptosis in cells, resulting in increased membrane density. The outer membrane ruptures while the nucleus is unaffected (19). The main basis for assessment of ferroptosis is whether lipid peroxidation occurs, thus, iron homeostasis and lipid reactive oxygen species (ROS) are the key factors for ferroptosis occurrence. GPX4 is a member of the GPxs family that can exert auto-antioxidant effects to suppress lipid ROS levels in cells during ferroptosis, thereby alleviating cellular ferroptosis (6).

The cystine-GSH-GPX4 axis is a major pathway against ferroptosis in mammals (20). Once inside the cell, cystine (Cys2) can be oxidized to cysteine (Cys), which is used to synthesize glutathione (GSH) in a reaction that is catalyzed by glutamate-cysteine ligase (GCL) and glutathione synthetase (GSS). Glutathione is the main antioxidant in ferroptosis and is synthesized from cysteine, glycine and glutamic acid (21). Glutathione (GSH) is an intracellular molecule that exists predominantly in its reduced state, allowing it to effectively scavenge ROS and protect cells from oxidative damage. However, when GSH encounters oxidizing substances such as ROS, it can become oxidized to form oxidized glutathione disulfide (GSSG). Using the electrons provided by NADPH/H+, oxidative glutathione (GSSG) is reduced to GSH by glutathione disulfide reductase (GSR), thereby creating a cycle (22). Enhanced GPX4-mediated reduction of any phospholipid hydroperoxides (PLOOHs) produced in cells results in production of corresponding alcohols (PLOHs).

Intracellular GSH levels are much higher than extracellular GSH levels, thus, they can participate in REDOX reactive drug delivery and are associated with tumor drug resistance (23). Cysteine plays a major regulatory role in this process. In mammalian cells, System Xc- introduces cystine (the oxidized form of cysteine) into the cell where it is used to generate gamma-glutamylcysteine ligase-mediated glutathione. For System Xc-, the main components include SLC3A2 and SLC7A11 fibers, which are embedded on cell membrane surfaces (24). The SLC7A11 plays a very important role as a subunit. It can promote cysteine transport and enable its transfer into cells for GSH synthesis. Downregulation of system XC-component SLC7A11 inhibits cystine uptake and suppresses the peroxidase activities of glutathione, thereby reducing the antioxidant capacities of cells and strengthening the sensitivity of cells to ferroptosis. Overexpressed SLC7A11 in human tumor cells inhibits ROS-induced “ferroptosis” (25). To prevent ferroptosis, cells rely on the antioxidant, glutathione (GSH), to neutralize PLOOHs. Essentially, GPX4 is a selenium protein that is a very important REDOX enzyme in mammalian cells. Physiologically, GPX4 reduces phospholipids and cholesterol peroxides to their corresponding alcohols, in a process that usually requires the presence of two electrons and selenocysteine residues that are provided by glutathione (18). Since GPX4 is the main PLOOHs neutralizing enzyme, erastin/Ras-selective lethal 3(RSL3) induces ferroptosis. The formation of ferroptosis is directly or indirectly correlated with inhibition of GPX4 through GSH depletion. Erastin indirectly inactivates GPX4-RSL3 by inhibiting cystine formation, thereby suppressing cysteine formation, a fundamental component of GSH in cells. Therefore, increasing the amounts of PLOOHs can result in very serious irreversible damage to the membrane, resulting in cell death (26). Inhibition of GPX4 can also cause tumor cell ferroptosis. Therefore, the typical GPX4 regulatory pathway plays an important role in tumor biology (27).

### 2.2 Phospholipid metabolism pathway

Increased lipid peroxidation is a classic feature of.ferroptosis As early as the 1950s, it was reported that selenium, vitamin E and cysteine have certain inhibitory effects on lipid peroxidation. Lipid peroxidation is trigger by the uptaking of hydrogen atoms located between two carbon-carbon double bonds in the lipid bilayer, resulting in the formation of a phospholipid radical at the center of the carbon chain. The phospholipid group reacts with oxygen to form phospholipid hydrogen peroxide radicals (PLOO) that obtain a hydrogen element from another PUFA to finally form PLOOH (28). If conversion is not performed according to the above process, GPX4, PLOOH, and lipid radicals [including phospholipid peroxide radicals (PLOO) and alkoxyphospholipid radical (PLO)] are not converted into their corresponding alcohols (PLOH), but the hydrogen atoms at the far end of PUFA are removed and reacted to form PLOOH, which forms PLOOHs with oxygen (29). These reactions produce various products that damage the cell membranes. Studies on neuronal cells have shown that higher levels of PUFA-PL in cell membranes are affected by peroxidation. However, it has not been fully established as to whether the membranes on the mitochondria, endoplasmic reticulum or corresponding enzymes are negatively affected by ferroptosis-induced lipid peroxidation. The unsaturation degree of lipid bilayers affects cell sensitivity to ferroptosis. Production of lipid or hydroxide radicals (OH) results in non-enzymatic lipid peroxidation reactions, which may be the result of side reactions (Fenton reaction) of the iron catalyst. Some lipoxygenases (LOXs), which are non-dependent heme dioxygenases, are targeted PUFAs (30). The enzyme can directly oxidize PUFAs and PUFA-containing lipids on biofilms. This implies that lipoxygenases (LOXs) can also cause ferroptosis. However, the involved mechanism is unclear. An association between lipid peroxidation and oxidative reductase (POR) has been reported in overexpressed cytochrome P450 (31). In the presence of POR, NADPH provides a large number of required electrons, and the levels of downstream electron acceptors (such as cytochrome P450, CYB5A) are reduced after receiving electrons. These outcomes are attributed to dehydrogenation of PUFAs or lipid peroxidation due to conversion of ferric iron to divalent iron (32). Therefore, it can be seen that iron and ferric iron have obvious effects on the Fenton reaction and lipid peroxidation process.

### 2.3 Iron metabolism pathways

Iron metabolic pathways are primarily dependent on iron, therefore, ferroptosis is an iron-dependent form of cell death. When ferroptosis occurs, there is an increase in levels of a small range of divalent irons, which is referred to as unstable iron pool phenomenon (LIP) (33). Fe^3+^ transferrin (TF) binds and is transported to tissues. Cell membranes have transferrin receptors (TFR). One molecule of TF can carry 2 molecules of Fe^3+^ to bind with TFR and enter the cell via endocytosis. Then, Fe^3+^ is released into the cytoplasm. Divalent iron levels in redox activities are key in assessing the production of PLOOH by Fenton reactions. The LIP is regulated by intracellular iron homeostasis. Typically, PLOOHs ionically react with ferric/divalent iron to form PLO and PLOO radicals, which lead to a series of peroxide chain reactions (32). Most of the ROS in cells is catalyzed by iron. Under catalytic actions of ROS, lipid peroxidation induces ferroptosis. Given the significance of iron in cell activity and ferroptosis, cellular iron homeostasis is achieved via iron-regulatory proteins (IRP1 and IRP2) (34). In the presence of unstable iron levels in cells, various cellular processes improve sensitivity to ferroptosis. For instance, degradation of ferritin by autophagy increases cell-to-cell free iron levels, which improves the utilization rate of iron in cells and activates ferroptosis. Transferrin (Tf) uses the circulation process to smoothly transport iron to cells, promoting ferroptosis. The mechanisms of action of cellular iron can effectively avoid ferroptosis. An association between iron obtained by degradation of heme produced by heme oxygenase 1 (HO-1) and ferroptosis has been reported. In these associations, HO-1 is effective in accelerating the iron sagging rate (35). Cancer cells require more iron to rapidly proliferate, thus, reducing intracellular iron levels is a novel approach for cancer treatment. Induction of ferroptosis is also a cancer treatment approach. Chemodynamic therapy (CDT) is an innovative cancer treatment strategy. The basic principle of CDT involves the use of nanozymes to activate intracellular fenton reactions to produce toxic hydroxyl radicals, thereby effectively removing cancer cells (36).

## 3 Ferroptosis and drug resistance of tumors

**Figure 2:**
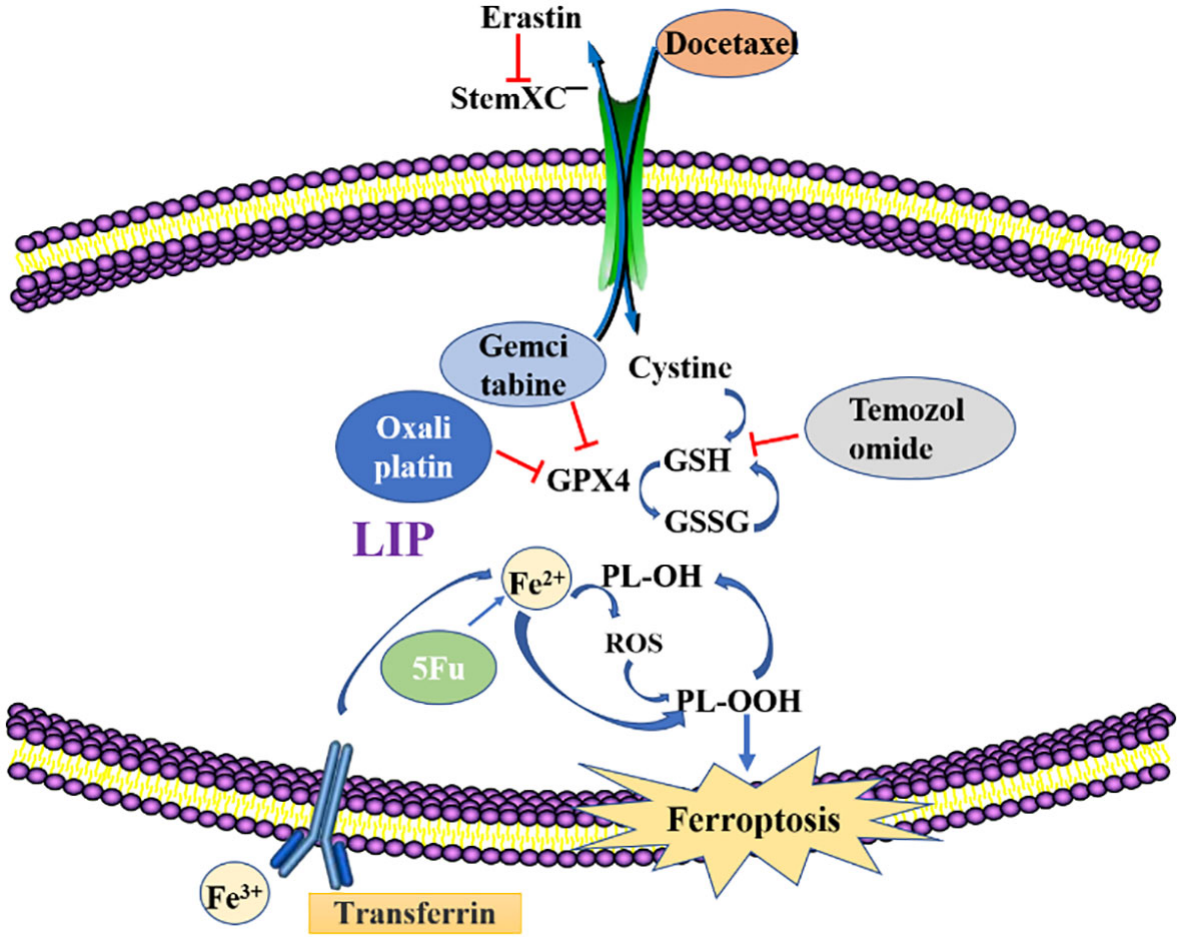
Methods to overcome resistance in ferroptosis. Erastin, Ferroptosis inducer; GSH, glutathione; GSSG, Glutathione(Oxidized); GPX4, recombinant glutathione peroxidase4; PLOOH, Phospholipid hydroperoxide; Fe^2+^/Fe^3+^, Ferrous ion; LIP, labile iron pool.

Programmed cell death is gradually enriched during the regulation of effector and immune cells. Immunosuppressive tumor microenvironments (Tmes) play a crucial role in determining the clinical outcomes of cancer therapies (37). Induction of ferroptosis in tumor cells is a feasible research direction in tumor therapy, and the compounds as well as mechanisms that induce ferroptosis in tumor cells will become the focus of research and development of antitumor drugs. Cancer cell drug resistance involves both intrinsic and acquired resistance mechanisms (38). Targeting ferroptosis is a potential strategy for overcoming drug resistance and improving the efficacy of anti-cancer treatments. Ferroptosis inducers can reverse acquired resistance of cisplatin, docetaxel, sorafenib and other drugs in tumors (39, 40). **Figure 2** and **Table 1** shows the roles of ferroptosis in overcoming drug resistance, strategies for overcoming cancer resistance and improving therapeutic efficacy by regulating cancer ferroptosis.

**Table 1:** Applications of ferroptosis in drug resistance in tumors.

Compound/Drug	Cancer type	Target	Function	References
Artesunate	Glioblastoma, Pancreatic cancer, Ovarian cancer	Ferritin(PDB ID: 5XHI)	Enhances lysosomal degradation of ferritin	(41–43)
Cetuximab	Colorectal cancer	GPX4	Inhibiting GPX4 activities	(44)
Cisplatin	Lung cancer, Colorectal cancer, NSCLC	GSH/SLC7A11	Induce Lipid peroxidation,increase the level of free iron, Activate the Nrf2/xCT pathway	(45, 46)
Cotylenin A	Pancreatic cancer	ROS	Promoting ROS production	(35)
Doxorubicin	Osteosarcoma, Ovarian cancer	GPX4(PDB ID: 2OBI)	Inhibition of xCT activity inhibits cystine and glutamic acid transport, thereby inhibiting GPX4 activity	(47, 48)
Everolimus	Renal cell carcinoma	VDAC2/VDAC3/GPX4(PDB ID: 2OBI)	Inhibition of xCT activities	(49)
Gemcitabine	Pancreatic cancer	GPX4	Inhibit lipid peroxidation	(50)
Gefitinib	Lung cancer, breast cancer	ROS GPX4	Inhibition of EGFR-ERK/AKT by gefitinib, reduces ROS	(51, 52)
Lapatinib	Breast Cancer, Glioblastoma	Fe	Induces ferroptosis by elevating the intracellular iron levels	(53)
Paclitaxel	Colorectal cancer	SLC7A11	Lipid peroxidation	(54)
Siramesine	Breast cancer	Iron	Increases iron	(53, 55)
Sorafenib	Glioblastoma,Liver Cancer,Colorectal cancer	SLC7A11	Induce lipid peroxidation, accumulation of ROS to promote ferroptosis	(56–58)
Statins	Lung cancer	GPX4(PDB ID: 2OBI)	Block the synthesis of GPX4	(59)
Sulfasalazine	Lung cancer, Glioblastoma, Colorectal cancer, Prostate cancer	SLC7A11 (PDB ID: 7CCS)	Inhibit cysteine uptake and GPX4 synthesis, promote ferroptosis	(45, 60–62)
Temozolomide	Glioblastoma	NRF2(PDB ID: 5CGJ)/ATF4(PDB ID: 6IRR)	Induce the expressions of GPX4, reduces GSH levels	(35, 63)

### 3.1 Ferroptosis in drug resistance

Induction of ferroptosis may be a new and effective approach for cancer treatment (64). Related drugs act on cancer cells and induce the release of large amounts of reactive oxygen species, resulting in ferroptosis. GPX4 and xCT are the most critical proteins for avoiding ferroptosis, thus, their inhibition can lead to ferroptosis in tumor cells and enhance cell death after conventional medical treatment. Blocking targeted xCT can effectively reduce cysteine uptake, leading to glutathione depletion and enhancing the sensitivity of tumor cells to chemotherapy. In addition, inhibition or loss of GPX4 can lead to the loss of lipid oxides, inducing ferroptosis (40). From these analyses, induction of iron fall significantly suppresses the hypermesenchymal cell status in tumor cells (65). We discussed the drug resistance performance as well as effects of anti-cancer drugs in iron fall, and proposed some solutions to overcome cancer drug resistance, particularly since the regulation of iron fall can significantly improve cancer treatment effects.

#### 3.1.1 Cisplatin

Cisplatin (DDP) is a very classic anti-cancer drug. It is used for treatment of bladder cancer, ovarian cancer, stomach cancer and other solid tumors. The mechanism by which DDP exerted anti-tumor effect is by binding to DNA and initiating cell death (66). Despite DNA damage, the signals promoting cell death are not sufficiently activated, which is partly due to downregulation of cell death and upregulation of signaling preventing cell death. DDP induces the overproduction of ROS, which is an important cytotoxic effect of the drug. Therefore, cisplatin induces ROS overproduction, which in principle may promote iron sag. ROS, lipid peroxides and MDA levels were lower in cisplatin resistant osteosarcoma cells, which may be related to the increase of antioxidant enzymes such as GPX4. Nrf2 is an important transcription factor that exerts its functions by binding to antioxidant response elements of several antioxidant enzymes and molecule. Cisplatin is able to increase the expression of Nrf2 by activating STAT3 signaling, and the overactivation of STAT3/Nrf2 signaling mediates the enhancement of antioxidant capacity of cisplatin resistant osteosarcoma cells. After MG63/DDP and Saos-2/DDP cells were exposed to cisplatin, the expression of p-STAT3, Nrf2 and GPX4 was higher than that of MG63 and Saos-2 cells. After the addition of the STAT3 inhibitor BP-1-102, the expression levels of Nrf2 and GPX4 in cisplatin-resistant osteosarcoma cells were significantly reduced. In addition, in the presence of STAT3 inhibitors, cisplatin notably decreased resistant cell viability by increasing ROS, lipid peroxides and MDA levels (67).

Inhibition and deletion of xCT depends on whether it induces ferroptosis, inhibits HNC cell apoptosis, and promotes cisplatin-resistant functions. Nrf2 is an important regulator of iron-promoting actions. Since Nrf2 binds Keap1, a degradation reaction occurs with ubiquitination in the proteasome, resulting in the loss of activities. Inactivation of the Nrf2-ANE antioxidant component pathway directly increases the sensitivity of HNC-resistant cells to artesunate, thereby reversing the resistance of drug-resistant cells to ferroptosis (68). Osteosarcoma cells resistant to cisplatin exhibit iron death inhibition after treatment with low dose of cisplatin, and are reactivated by inducing iron death inducers. The combination of cisplatin drugs and iron dip inducers increases the sensitivity of resistant cells to cisplatin (67). Cisplatin and paclitaxel activated tumor-associated fibroblasts (CAFs) in gastric cancer cells to secrete miR-522, which inhibited tetraenoate lipoxygenase 15 (ALOX15), thereby reducing the accumulation of lipid peroxidative ROS, inducing ferroptosis (69), and achieving drug resistance. Therefore, inducing ferroptosis can significantly inhibit the drug resistance effects that are attributed to the combination of cisplatin and paclitaxel. ATF3 induces cancer cell ferroptosis by blocking various signaling pathways (e.g., Nrf2, Keap1, and xCT among others), thereby reducing cisplatin resistance in gastric cancer cells (40). The cisplatin-induced Nrf2 and xCT signaling pathways differ in different NSCLC cell lines, and the degree of activation is closely associated with cisplatin resistance level. The Nrf2 and xCT signaling channels exhibited significantly increased cell levels of cisplatin-resistant NSCLC. In non-small cell lung cancer (NSCLC) cell lines, cisplatin induces both apoptosis and ferroptosis. In the cytoplasm, cisplatin drugs bind GSH to form a Pt-GS complex structure. The structure is similar to Erastin. Therefore, suppression of GSH levels and GPX inactivation are the mechanisms of cisplatin-induced ferroptosis (70). The GPX4 inhibitors are effective in all tumors, implying an association between targeted therapy modalities for tumors and GPX4 inhibitors (71). However, the combination of multiple drugs can produce toxic effects. Increasing the levels of GPX4 inhibitors has been shown to sharply increase the oxidative sensitivity of drug-resistant cancer cells, and the time period involved is very long. Thus, targeted therapy or chemotherapy can be completed within that time. The new treatment regimen with staggered time may achieve great results in clinical treatment and reduce its possible toxic side effects. Thus, GPX4 inhibitors are able to increase the therapeutic efficacy of platinum-based drugs against drug-resistant tumor cells (72).

#### 3.1.2 Docetaxel

Docetaxel is a semi-synthetic paclitaxel that is widely used as an antitumor drug. Its effects are comparable to those of paclitaxel and it can treat more tumor types.

Docetaxel is a phase M period-specific drug that promotes tubule polymerization into stable microtubules and inhibits their polymerization, thus significantly reducing the number of tubules and destroying the microtubule network structure, leading to the formation of stable non-functional microtubules, thus disrupting the mitosis of tumor cells (73). It can be used alone or in combination with other chemotherapy drugs. Despite the significant anti-cancer effects of docetaxel, its resistance remains a major challenge in clinical application. Overexpression of ABCB1 is associated with multidrug resistance (MDR) in tumor cells. Up-regulation of ABCB1 is the reason for the docetaxel resistance in patient with ovarian cancer with poor survival. Erastin is a novel small molecule targeting SLC7A11 induced iron sag. In ABCB1 overexpressed ovarian cancer cells, erastin co-delivery with docetaxel significantly reduced cell viability, promoted apoptosis, and induced cell cycle arrest at G2/M. Mechanically, erastin improves intracellular ABCB1 substrate levels by limiting the drug effector activity of ABCB1, without altering ABCB1 expression. erastin can therefore reverse ABCB1-mediated docetaxel resistance in ovarian cancer (74). Artesunate (ART) (dose - and time-dependent) significantly limited cell growth and proliferation in parental and docetaxel-resistant PCa cells, but had no effect on “normal, non-cancerous” cells. In all Docetaxel-resistant PCa cells and parental LNCaP, ART-induced inhibition of proliferation was associated with G0/G1 phase arrest and down-regulation of cell cycle activating proteins. ART also promotes apoptotic cell death in parental and docetaxel-resistant PC3 and LNCaP cell lines. ART induces apoptosis of parental and DX-resistant DU145 cells by increasing ROS generation (75).

As a substandard alternative to paclitaxel chemotherapy, it is highly demanded in the national oncology drug sales market (76). The medicine can be used alone or in combination with other drugs such as paclitaxel. Microtubules play an important role in cell mitosis. Docetaxel interferes with cell structures of microtubules to deregulate their effects, thereby inhibiting tumor cell mitosis. Despite its evident anti-cancer effects, this drug is associated with various clinical challenges. The small molecule Erastin, which can induce ferroptosis, significantly reduces SLC7A11 levels, thereby inhibiting cystine uptake, resulting in reduced GSH synthesis. Low levels of Erastin, an iron-dead inducing substance, were able to strongly downregulate SLC7A11, thereby blocking cystine transport, leading to GSH depletion. Erastin is involved in reversal of ABCB1-induced docetaxel resistance in ovarian cancer. Erastin and docetaxel reduce cell activities, promote apoptosis, and are effective tumor treatment plans. The xCT inhibitor, sulfamezosin (SAS), has certain effects on paclitaxel-resistant efficacy in the uterine serous cancer cell system, and its damage to paclitaxel-resistant cells is greater than in other sensitive cells, therefore, it induces ferroptosis rather than apoptosis (74).

#### 3.1.3 Gemcitabine

Gemcitabine (GCB) is phosphorylated by dioxycytidine kinase (DCK), when it was internalized via nucleic acid transport. The phosphorylation leads to the formation of GCB-triphosphate, which is able to inhibiting nuclear replication by incorporating into cellular DNA (77). Gemcitabine is a pyrimidine analog that can be doped into replicating DNA to indirectly inhibit the DNA synthesis process. When GCB enters the human body, it is converted into various active drugs. Metabolites of this drug have significant inhibitory effects on DNA synthesis. Even though gemcitabine has significant therapeutic efficacies in treatment of some cancer patients, its clinical applications are limited by resistance. Its mechanism of action is influenced by various enzymes, therefore, occurrence of drug resistance is associated with several factors. The basic chemotherapy agent used for pancreatic ductal adenocarcinoma (PDAC) is GCB, and a strong association between gemcitabine and inducers of ferroptosis has also been found after studying the RNA sequences of tumor samples (78). We found elevated GPX4 levels in gemcitabine-resistant PDAC cells. Mechanistically, transcription factor 4 (ATF4) activation induces HSPA5, which binds glutathione peroxidase 4 (GPX4) to prevent its degradation and subsequent lipid peroxidation. Outside the cell body, drug inhibition increases sensitivity between gemcitabine and ferroptosis (59). The sensitivity of PDAC patients to ferroptosis is positively correlated with responses of highly infiltrating CD8+ T cells and type II interferons (79). CBR1 acts on cells to protect them from oxidative stress and its suppression regulates ROS levels, thereby inhibiting PCA cell proliferation. Suppressed CBR1 levels promote gemcitabine susceptibility. Flavonoid albumin directly binds CBR1 and inhibits the activities of related enzymes, increasing free iron levels in cells, cell ferroptosis and gemcitabine sensitivity (80).

#### 3.1.4 Temozolomide

Temozolomide (TMZ) is a common chemotherapeutic drug that is used to treat oncological diseases, such as breast and bladder cancers.

It alkylates genomic DNA at the N7 and O6 sites of guanine and N3 sites of adenine and induces nucleotide mismatch during the subsequent replication cycle (81). Nucleotide mismatch causes cytosine to be replaced by thymine, as opposed to methylated guanine. Mismatch repair recognizes small nucleotide insertions/deletions that promote cell cycle arrest in the G2/M phase, leading to tumor cell death. DNA repair activity is able to regulate the sensitivity of cytotoxic chemotherapy drugs. Overactivation of O6-methylguanine-DNA methyltransferase (MGMT) by removing the alkylation of different nucleotides induced by tmz is the most important factor in the development of tmz resistance in GBM (82).

Temozolomide is not overly inhibited by drug resistance. The GSCs are a kind of cell community in glioma tissues. After TMZ chemotherapy, glioma cells were partially converted into GSCs, which increased resistance to TMZ. The TMZ-resistant glioma cells have elevated ROS and DMT1 expressions, compared to sensitive cells, implying decreased GPX4 levels inside cells and occurrence of ferroptosis (83). When the erastin cystine conversion process in the Xc-system was blocked, the drug-resistant transcription factor Nrf2 promoted the expressions of targeted ABCC1. This could be a breakthrough in reversing glioma resistance (84).

#### 3.1.5 Oxaliplatin

Oxaliplatin enters tumor cells via copper transporter 1 (CTR1), where it goes through the activation step of chlorine ligand substitution, usually replaced by water molecules or other small molecules containing sulfhydryl groups. Oxaliplatin is more stable to hydration due to chelation of departing ligands. Oxaliplatin binds to DNA through the formation of intra-strand and inter-strand crosslinks, changes DNA structure, and causes DNA damage (85). The most nucleophilic DNA site is the N7 site of guanine, which is exposed to the main groove and preferentially binds to oxaliplatin. This kind of DNA damage can halt the cell cycle and induce apoptosis in of tumor cells. Glutathione is one of the most abundant non-protein mercaptans in tumor cells, and it is the most important intracellular mercaptan compound involved in the mechanism of cell drug resistance (86). Cancer cells can use endogenous GSH to chelate Pt drugs, producing inactive GSH-PT adducts that can be preferably pumped out by membrane transport, increasing drug resistance to cancer cells (87).

Platinum drugs play a significant role in precision treatment of cancer and related immune therapy. However, their applications are limited by drug resistance. Platinum drugs bind the DNA of tumor cells to form Pt-DNA complexes, thereby affecting the subsequent DNA transcription process and inducing tumor cell death (88). Excess accumulation of platinum drugs in the body results in drug resistance. The mechanisms involved in platinum drug resistance include reduced platinum levels in cells, platinum inactivation, improved DNA repair abilities, and increased degree of inhibition of tumor cell apoptosis. Oxidative stress based on oxaliplatin enhances the phosphorylation level of NFS1 serine residues, thereby preventing PAN apoptosis in a S293 phosphorylation dependent manner during oxaliplatin treatment (89).

#### 3.1.6 5-FU

Fluorouracil (5-FU) is considered to be the most effective antitumor agent for the treatment of advanced colorectal cancer. Drugs need to be converted to nucleotide levels in order to work. It can be incorporated into RNA, which is able to inhibit the maturation of nuclear RNA. However, its conversion to 5-fluoro-2 ‘deoxy-5’ monophosphate (FdUMP) leads to the inhibition of thymoadenylate synthetase (TS) and subsequent DNA synthesis, which is thought to be its primary mechanism of action. In the presence of folic acid cofactors, a covalent ternary complex is formed to achieve its stability. 5-FU can be inactivated by degradation to 5-fluordihydrouracil (F-DHU). It is further degraded to 5-fluoro-alanine (F-BAL), which can be further metabolized. Beta-alanine itself is a substrate for carnosine, but it can also be converted to acetate. Similarly, F-BAL can be converted to fluoroacetic acid, which is associated with neurotoxicity. 5FU degradation occurs in all tissues, but tumor tissues contain very small amounts of dihydroouracil dehydrogenase. Although the activity of this enzyme occurs in the kidneys, it is strongest in the liver, which means that the liver plays an important role in the degradation of 5-FU (90). 5-FU is a common anti-cancer drug with significant inhibitory effects on many tumors. It acts in the S phase. After its transformation in the human body, 5-fluorouracilin (5-FUR) is formed, which interferes with the RNA protein synthesis process. Therefore, this drug has an effect on tumor cells in all stages. A recently published article reported the exact mechanism of resistance to 5-FU, which involve a common BOK protein. The BOK acts as a regulator of multiple cellular processes *in vivo*, can bind UMPS enzymes to participate in DNA synthesis and plays an important regulatory role in cell proliferation. In drug chemotherapy, the UMPS enzyme metabolizes 5-FU into a toxic metabolite (5-FUMP), which enters the proliferating cells, poisoning them and inhibiting their proliferation (64).

### 3.2 Ferroptosis in tumor cells

There is a strong link between ferroptosis and cancer. The ferroptosis-inducing compounds are gradually being discovered in the search for new ways to treat cancer. Most of the signaling pathways associated with cancer are regulated by ferroptosis. The mesenchymal epithelial cells have some resistance to apoptosis and common therapeutic drugs, however, they are sensitive to regulation of ferroptosis. Ferroptosis, a form of cell death that is caused by oxidative overreactions, is associated with metabolic processes of cells. During *in vivo* growth, cells have high metabolic activities and ROS levels (4), therefore, determination of ferroptosis is also reasonable. Tumor cells often require an environment with higher iron levels, implying that cancer cells are more sensitive to iron fall. Tumor cells can also use their own genetics to combat metabolic processes in the body. For instance, many tumor cells are more sensitive to ferroptosis when SLC7A11 or antioxidant transcription factor Nrf2 levels are elevated. Thus, induction of ferroptosis in different cancer types is depended on the genetic background of the tumor type.

The inhibitory factors secreted by tumor cells are more sensitive to cells and ferroptosis. p53 is a tumor suppressor whose activation can reduce SLC7A11 expressions during RNA transcription and protein synthesis. Inhibition of SLC7A11 expressions suppresses SystemXc- activities and leads to ferroptosis (7). Moreover, p53 has polymorphism characteristics, which may lead to replacement of P47S amino acids. The association between the loss of iron decay activities and specific genetic mutations has yet to be established. The BAP1 regulator, which is similar to p53, plays a certain role in regulating SLC7A11 expressions (91). The difference between them is the degree of effective inhibition, which should be investigated further. Fumarate enzyme plays an important role in the TCA cycle by catalyzing the formation of malic acid, a major inhibitory molecule for leiomyoma and renal papillary cell carcinoma. However, it has not been determined how much energy malic acid has and how it inhibits metabolic processes. The fumarate enzyme also accelerates ferroptosis, which is closely associated with mitochondrial ROS synthesis (17). If fumarate enzyme is inactivated, then, oxidation reactions that occur inside the mitochondria will be severely affected.

Cancer cells are more sensitive to ferroptosis. Many factors, such as deregulated cancer cell proteins and signaling pathways affect the occurrence of iron fall disease. Through these changes, cancer cells can be alerted to the degree of response induced by iron falling. Using the E-cadherin NF2 Hippo YAP signaling pathway as an example, E-cadherin loss-of-function causes a range of mutations in cancer, with NF2 loss-of-function mutations occurring in more than three-tenths of mesothelioma (91, 92). Mutations in the YAP gene are rarely observed in signaling pathways, however, traces of expressions of this gene are often detected (93). Mutations cause cancer cells to become more sensitive to iron fall. The involved biomarker signals should be determined.

## 4 Epithelial–mesenchymal transition (EMT) in tumors and drug resistance

There are two important cell species in vertebrates, mesenchymal cells and epithelial cells, both involved in epithelial-mesenchymal transition (EMT). After EMT, some cells retain most of the characteristics of epithelial cells, but also acquire some of the properties of mesenchymal cells while some cells are completely transformed into mesenchymal cells. There are many factors that induce the EMT process, including cellular transcription factors, oxidative stress responses, hypoxia, and conduction between cell signals.

### 4.1 Tumor and EMT

The EMT process is a major driver of cancer cell development from the initial stages to advanced cancer stages. This process plays a role in multiple tumorigenic processes. For instance, it reduces cell surface adhesion, enhances cell invasion, antioxidant effects and drug resistance outcomes among others (94). Tumor malignancy is associated with EMT. During EMT, E-cadherin expressions exhibit a significant downward trend while N-cadherin expressions are relatively increased, which is the core step of EMT. E-cadherin is located inside cells, is a marker of epithelial tissues and is responsible for adhesion between cells. However, N-cadherin presents a weak connection in mesenchymal cells, leading to cancer cell metastasis and invasion after EMT. Moreover, EMT results in production of an intermediate fibrin structure, which is the main cause of cellular flexibility (95). Vimentin levels in breast and prostate cancers are significantly increased. In a patient with advanced breast cancer, vimentin levels were found to be significantly increased, which was attributed to regulation of Smad interacting protein 1 (SIP1) (96). Apart from the above mentioned waveform proteins, there are many mesenchymal markers that epithelial EMT can use (97).

Transcription factors such as Snail, Zeb1, Slug, Twist, and FOXC1 can also act as inducible factors in EMTs, implying that they are potential targets for cancer treatment (98). Snail is an important EMT inducer that can inhibit E-cadherin gene expressions (99). Zeb1 binds histone deacetylase Sirt 1 and E-cadherin targets, thereby suppressing E-cadherin gene expressions (100). In addition, Zeb1 expressions in bronchial epithelial cells results in increased levels of a variant mesenchymal, CD44 (101). In cholangiocarcinoma Snail increases Sp1 expressions in the presence of PKC, inducing EMT (102).

### 4.2 The relationship between tumor EMT and drug resistance

The EMT process results in a loss of cellular adhesion in epithelial cells, a property that is characteristic of fibroblasts. The transformation targets cancer stem cells to increase tumor cell migration. Moreover, EMT can lead to cell resistance. Transcription factors such as Zeb1, Snai1 and Twist1 regulate and promote EMT. Thus, they are potential factors for tumor cell metastasis. Since cells lose their original epithelial characteristics and acquire some mesenchymal features, then, EMT in cancer cells increases metastasis, invasion and drug resistance rates (103).

Hypothetically, EMT-induced transcription factor expressions affect the degree of acquired resistance. Single-cell studies revealed EMT and some resistant cohort cells in primitive cancer cell communities that had not been exposed to drugs (104). The ABC transporter is a very large transmembrane transport protein that plays an important role in activities of many organisms. During the transcription of ABC transporters, many promoters are linked to EMT-induced transcription factors, and drug resistance is acquired while activating EMT. Induction of EMT-associated transcription factors increases resistance to apoptosis, which is distinguished from the resistance mechanism of ABC transporters (105). Detection of EMT-induced transcription factors in tumor cells is an important marker for determining sensitivity or resistance.

Snail is a zinc-finger transcription factor that inhibits the transcription of cadherin in epithelial cells while slug is a widely expressed transcriptional suppressor protein. They can both inhibit transcription. They influence EMT by controlling epithelial gene expressions. Snail regulates p-glycoprotein, MDR1 as well as BCRP expressions, and excretion of p-glycoprotein active drugs from the body. Both zinc finger transcription factors are capable of inducing resistance, independently of the ABC transporter (106). Snail affects cyclin D2 transcription, indirectly harms the normal cell cycle and enhances resistance to both endogenous and exogenous-related apoptotic pathways (107). When EMT occurs, elevated Snail levels can also cause metabolic changes in cancer cells. Increased uptake of nutrients such as glucose by cells, increased lactic acid production, and decreased mitochondrial activities increase cell resistance to chemotherapy (108). The Twist protein is an evolutionarily consistent basic helical cyclic transcription factor that acts as a key mesenchymal regulator during cellular embryo development. In many cancer types, the levels of this protein are increased, which is associated with adverse reactions and invasive abilities. Twist1 increases the resistance of P-gp inhibitors and P-gp inducers to P-gp substrate drugs. It can alter P-gp expressions, resulting in elevated levels of other types of ABC transporters (109). Since both Twist and Snail suppresses the expressions of nucleoside transporters, they can induce resistance to nucleoside analogues. In mice assays, ENT 1 and CNT 3 levels were significantly increased in PDAC mice lacking the Twist1 or Snaill genes (110). Both Twist and Snail are expressed in human tumor cells. In breast cancer, suppressed Snail2 expressions are dependent on Twist1 levels. Since Twistl increases AKT2 gene expressions levels in primitive tumor cells, the cell survival signaling pathway is improved, which reduces paclitaxel drug sensitivity (111). The ZEB1 protein is a key transcription factor in inducing EMT and cell plasticity in cancer cells. In breast cancer cells, Zebl enhances the expressions of multiple ABC transporters *in vitro* (112). This protein protects cells from stress toxicity and facilitates checkpoint kinase 1 (CHK1) to monitor DNA damage during DNA replication (113).

## 5 Crosstalk between ferroptosis and EMT

**Figure 3:**
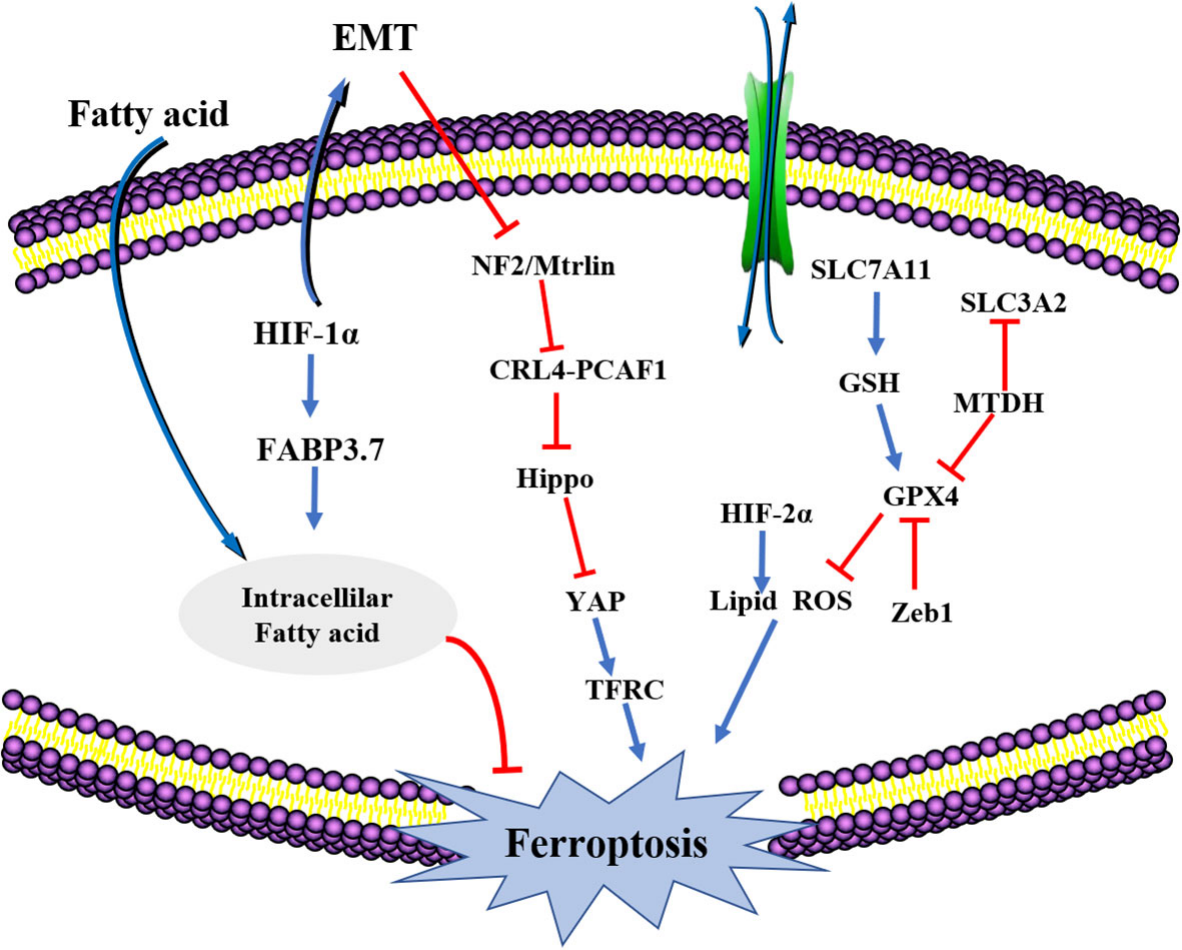
The crosstalk between EMT and ferroptosis. HIF-1a, hypoxia-inducible factor-1 (HIF-1); FABP3.7, fatty acid binding protein 3; NF2, Neurofibromatosis type II; SLC7A11, solute carrier family 7, (cationic amino acid transporter, y+ system) member 11; HiF2a, Recombinant Hypoxia Inducible Factor 2A (HIF2A); SLC3A2, solute carrier family 3 (activators of dibasic and neutral amino acid transport) member 2; GPX4, Recombinant Glutathione peroxidase4; Zeb1, Zinc Finger E-Box Binding Homeobox 1.

EMT-mediated tumor cells have drug resistance properties and interstitial resistant cancer cells are prone to ferroptosis. There is a complex relationship between ferroptosis and EMT that is mediated by multiple signaling pathways (114). Ferroptosis is a potential approach for reversing or inhibiting EMT and thus reversing drug resistance (**Figure 3**).

The higher degree of mesenchymal cell states has been attributed to transmission and actions of the GPX signaling pathway. Therefore, regulation of GPX4 levels inside cells induces cancer cell ferroptosis, thereby blocking further spread and metastasis (115). The MET adhesion protein (MTDH) is a newly discovered cancer-related functional protein that is responsible for many types of EMT transformation, cell invasion and metastasis. Suppressed MTDH levels inhibits GPX4 and SLC3A2 expressions, impacting the synthesis of cysteine and GSH, and indirectly affecting glutamate levels, enhancing the sensitivity of cells to ferroptosis (116). Increased levels of transcription factors like Snail, Twist1 and Zeb1 enhances the sensitivity of tumor cells to ferroptosis. Zebl, a transcription factor involved in cell transfer that works by regulating GPX4 activities, is affected by EMT (117). Moreover, Zebl induces PPARγ expressions, whose activation induces the apoptosis of tumor cells through different pathways. Zeb1 affects cellular lipid metabolism to effectively regulate the uptake of lipid substances, their accumulation, and the EMT-associated plasma membrane remodeling. The remodeling process is mainly located at the PUFA oxidation site (118). Therefore, multiple EMT transformation process-associated factors increases tumor cell sensitivity and ferroptosis. When EMT occurs, the first thing is to disconnect epithelial cell connections. In epithelial cells, interactions between E-cadherin influences and inhibits the occurrence of ferroptosis with the help of NF2 and Hippo signaling pathways. This may be the mechanism by which EMT affects ferroptosis (119).

The lipid-associated metabolic challenges or lipid peroxidation can affect ferroptosis and trigger EMT. Overexpressed lipid-metabolizing enzymes can directly affect EMT occurrence. Disease symptoms associated with long-chain fatty acid ligase ACSLs affect lipid metabolism processes *in vivo* and play a role in cell proliferation, differentiation and apoptosis (120). During EMT conversion, activated Zeb1 increases the synthetic levels of PUFAs, which increases the levels of active lipid peroxides, enhancing the sensitivity of tumor cells to ferroptosis. Metabolic processes of transformed molecules such as GPX4 and PUFAs protect against cellular lipid peroxidation processes and indirectly induce ferroptosis (117).

Increased oxidative stress levels within cells, increased lipid peroxidation, and suppressed SLC7A11 levels are responsible for inducing ferroptosis and EMT conversion. TGF-β1 can make the apoptosis and EMT transformation processes to occur at the same time, that is, in the same type of cells. Fe-1 affects TGF-β1-induced EMT conversion and reverses erastin-induced ferroptosis (121). Therefore, TGF-β1 has certain delayed effects on ferroptosis occurrence, but has no preventive effect. The levels of GPX4 do not affect TGF-β1-induced EMT expressions and the degree of erastin-induced ferroptosis. After successful induction of EMT by TGF-β1, FTH1 expressions inside the cell decrease, while LIP and ROS expressions increase (122). These findings imply elevated iron levels and high levels of oxidative stress in tumor cells undergoing EMT, leading to ferroptosis. Increased expressions of E-cadherin is associated with activation of Merlin and Hippo signaling pathways. The reprogramming process for EMT plasticity is involved in the regulation of several important transcription factors (TFs), the most classic of which are transcription factors such as Snail, Zeb1, and Twist, which influence each other. The epigenetic reprogramming process of EMT induces SIRT1 or reduces miR-200 to achieve negative feedback inhibition. This triggers EMT to increase the sensitivity of iron sagging *in vivo* and *in vitro*. In addition, miR-200 suppresses E-cadherin levels, leading to frequent EMT conversion, thereby promoting cancer development. Overexpressed ZEB1 increases sensitivity to ferroptosis (123). Increased levels of Snail, Twistl, and Zeb1 transcription factors protects cells from ferroptosis (124). LYRIC is a functional protein that regulates EMT and its activation involves the activation of transcription factors (YAP1 and WWTR1/TAZ) of the Hippo signaling pathway, inducing tumor cell ferroptosis (125).

Selective non-expressions of SIRT3 gene increases GSH as well as NADPH levels in GBC and suppresses ROS levels. The SIRT3 gene affects various enzymes, primarily catalase and SOD2, both of which regulate ROS levels (126). The specific molecular mechanisms by which ROS is depleted are unclear, however, the decline in lipid peroxidation levels alleviates ferroptosis. SIRT3 can also AKT-dependently regulate EMT. Physiologically, AKT affects different carcinogenic signaling pathways in tumor cells (127). These signaling pathways can protect tumor cells from ferroptosis or EMT. Thus, in cells with suppressed SIRT3 expressions, the AKT signaling pathway is a potential therapeutic target (126).

β-elemi combined with cetuximab induces ferroptosis in tumor cells and inhibits EMT transformation. TGF-β1 is a versatile protein that regulates multiple cell types. Flavonoids (GNA) can inhibit TGF-β1-induced anti-melanoma. Ferritin functions as an integrator in GNA, activating p53, SLC7A11, and GPX4 signaling pathways and promoting ferroptosis in TGF-β1-induced cell EMT transformation (128). Upregulated ZEB1 expressions and downregulated FTH1 and SOD/GSH antioxidant enzyme levels increases lox-induced PUFA oxidation levels, resulting in ferroptosis in melanoma cells and inhibiting the EMT process (129). β-allene can be isolated from turmeric solution, and is often used in combination with targeted drugs. Colorectal cancer (CRC) is caused by continuous stimulation of the signaling pathway of the KRAS protein. The combination of β-lamimycin antibiotic and cetuximab can efficiently increase iron-dependent ROS levels, resulting in decreased lipid peroxidation and GSH levels. This treatment modality prevents cell invasion by regulating the interstitial marker (MMP-9) and increasing E-cadherin levels to prevent EMT from occurring (122).

MPC1 is a mitochondrial membrane protein that is affected by KDM5a. Since the KMPC1 signaling pathway in KDM5a is involved in cancer cell proliferation and metastasis, suppressed MPC1 levels may induce EMT. This decreases the KDM5A levels of ErPCC cells, increases MPC1 levels and induces ferroptosis. Therefore, the KDM5A-MPC1 axis may have therapeutic effects on HNC tumor cells (130).

Although most cancers are eliminated by drugs, some resistant tumor cells and cancer stem cells survive because of their drug-resistant properties. Ferroptosis may be used to kill the drug-resistant cancer cells. Decreased expressions of target genes for Nrf2 suppresses GPX4, GSH, and NADPH levels, resulting in cell lipid peroxidation and ferroptosis (131). Treatment of drug-resistant cells involves the EMT transformation process, which is removed by mesenchymal transformation into an inducer of ferroptosis.

## 6 Conclusions and prospects

Ferroptosis is fundamentally different from traditional forms of cell death like apoptosis, necrosis, and autophagy. It is initiated by the loss of activity of glutathione peroxidase, leading to impaired lipid metabolism and resultant cell death. This pathway’s specificity lies in its reliance on iron, making it unique and significant in the context of cancer, where iron metabolism often goes awry. Tumor cells exhibit altered iron regulation, which is intricately linked to the pathophysiology of cancer, including tumor metastasis, development, and particularly, drug resistance.

The connection between ferroptosis and EMT, a process by which epithelial cells lose their cell polarity and adhesion to become more mobile mesenchymal cells, is particularly noteworthy. EMT is a critical factor in cancer progression and metastasis, and its involvement in drug resistance has been increasingly recognized. The review highlights how ferroptosis and EMT intersect, influencing each other and contributing to the complexity of cancer biology.

The resistance of cancer cells to ferroptosis could be attributed to several factors, including genetic mutations, altered signaling pathways, and changes in the tumor microenvironment. The role of key proteins like GPX4 in maintaining the balance between oxidative stress and antioxidant defenses is crucial in this context. The GPX4 enzyme acts as a bulwark against lipid peroxidation, and its inhibition could trigger ferroptosis. Conversely, the upregulation of GPX4 has been linked to increased resistance of cancer cells to ferroptosis, presenting a potential target for therapeutic intervention.

Simultaneously, the EMT process contributes to the acquisition of drug resistance in tumor cells. EMT-induced transcription factors like Snail, Zeb1, and Twist have been shown to regulate and promote EMT, thereby contributing to tumor cell metastasis. The review discusses how these factors influence the sensitivity of cancer cells to ferroptosis, creating a feedback loop that affects cancer progression and treatment outcomes.

As we described in this paper, a connection is emerging between ferroptosis, EMT, and resistant mediators in the progression of tumor, but further research are needed to explore the mechanisms underlying this interaction. The interaction between resistance mediated ferroptosis and EMT may allow the development of potential therapeutic strategies (i.g. ferroptosis inducers or EMT inhibitors) to eradicate cancer cells by overcoming resistance. For example, several clinical trials are currently underway using anti-cancer drugs with iron-induced death activity, namely sulfasalazine for glioblastoma patients (NCT04205357) and Sorafenib for neuroblastoma patients (NCT02559778). TACIMA-218, a novel bioactive anticancer compound, leads to the destruction of REDOX homeostasis and key metabolic pathways, inducing tumor cell death (132). PARP inhibitors (PARPi) have a strong synergistic effect with type I PRMT inhibition. The combination of MS023 and the PARP inhibitor BMN-673 (talazopanib) has shown a strong synergistic effect in small cell lung cancer (133). Tetrahydroβ-carboline scaffold compound (YH677). YH677 inhibited epithelial mesenchymal transformation (EMT) and stem cell marker expression in a dose-dependent manner (134). A novel NAMPT inhibitor (A4276) selectively targets NAPRT-deficient EMT subtype cancer cells and alleviates chemotherapy-induced peripheral neuropathy (135). When considering the treatment of cancer with ferroptosis, it is of great significance to rationally design the combination of drugs to achieve the synergistic anticancer effect.

In addition, more efforts are needed to investigate the targeting mechanism of ferroptosis with EMT, viable clinically targeted methods for ferroptosis, and optimized synergistic combined ferroptosis cancer therapies to successfully translate this approach into the clinic. However, there is still a long way to go before these treatments can be used in practice, and inevitable challenges remain. Firstly, compounds that induce ferroptosis activity can induce and promote ferroptosis to overcome chemotherapy resistance; However, the exact molecular mechanism by which these compounds induce ferroptosis is not fully understood. Secondly, ferroptosis may play an important role in a variety of diseases in addition to tumors. Such as neurological diseases, cardiovascular diseases, inflammatory diseases, kidney diseases and lung diseases. Treatment to induce ferroptosis can be a double-edged sword. Therefore, the treatment of iron-mediated cell death needs to be carefully considered. There is still an urgent need to develop predictive biomarkers that mark ferroptosis *in vivo* to accurately predict tumor response to ferroptosis induction.

In conclusion, ferroptosis has been recognized as a key cell death response caused by chemotherapy for various cancers. Ferroptosis and EMT play an important role in the occurrence and development of tumors. The study of the molecular mechanism and interrelationship between ferroptosis and EMT is expected to provide new targets and strategies for tumor therapy and promote the progress of tumor therapy. However, the current research on the role and relationship between ferroptosis and EMT in tumors is still in the initial stage, and further studies are needed to reveal its specific mechanism and application prospects.

## Author contributions

HMZ: Writing – original draft, Writing – review & editing. NC: Writing – original draft, Writing – review & editing. CD: Writing – original draft. HNZ: Writing – original draft. DL: Writing – review & editing. SL: Writing – original draft, Writing – review & editing.
